# Combination of Dissolving Microneedles with Nanosuspension and Co-Grinding for Transdermal Delivery of Ketoprofen

**DOI:** 10.3390/ph16030378

**Published:** 2023-03-01

**Authors:** Delly Ramadon, Fathin Ulayya, Annisa Sakinah Qur’ani, Iskandarsyah Iskandarsyah, Yahdiana Harahap, Qonita Kurnia Anjani, Vania Aileen, Pietradewi Hartrianti, Ryan F. Donnelly

**Affiliations:** 1 Faculty of Pharmacy, Universitas Indonesia, Depok 16424, Indonesia; 2 Faculty of Military Pharmacy, Republic of Indonesia Defense University, Bogor 16810, Indonesia; 3 School of Pharmacy, Medical Biology Centre, Queen’s University Belfast, 97 Lisburn Road, Belfast BT9 7BL, UK; 4 School of Life Sciences, Indonesia International Institute for Life Sciences, Jakarta Timur 13210, Indonesia

**Keywords:** co-grinding, dissolving microneedles, ketoprofen, nanosuspension, solubility

## Abstract

Ketoprofen is an anti-inflammatory agent that may cause gastric irritation if administered orally. Dissolving microneedles (DMN) can be a promising strategy to overcome this issue. However, ketoprofen has a low solubility; therefore, it is essential to enhance its solubility using certain methods, namely nanosuspension (NS) and co-grinding (CG). This research aimed to formulate DMN containing ketoprofen-loaded NS and CG. Ketoprofen NS was formulated with poly(vinyl alcohol) (PVA) at concentrations of 0.5%, 1%, and 2%. CG was prepared by grinding ketoprofen with PVA or poly(vinyl pyrrolidone) (PVP) at different drug–polymer ratios. The manufactured ketoprofen-loaded NS and CG were evaluated in terms of their dissolution profile. The most promising formulation from each system was then formulated into microneedles (MNs). The fabricated MNs were assessed in terms of their physical and chemical properties. An in vitro permeation study using Franz diffusion cells was also carried out. The most promising MN-NS and MN-CG formulations were F4-MN-NS (PVA 5%-PVP 10%), F5-MN-NS (PVA 5%-PVP 15%), F8-MN-CG (PVA 5%-PVP 15%), and F11-MN-CG (PVA 7.5%-PVP 15%), respectively. The cumulative amounts of drug permeated after 24 h for F5-MN-NS and F11-MN-CG were 3.88 ± 0.46 µg and 8.73 ± 1.40 µg, respectively. In conclusion, the combination of DMN with nanosuspension or a co-grinding system may be a promising strategy for delivering ketoprofen transdermally.

## 1. Introduction

Inflammation is the body’s defense mechanism to eliminate the cause of trauma or infection in cells and start the cell repair process [1]. Inflammation of the joints is called arthritis. Osteoarthritis is a joint disease with a relatively high prevalence [2]. Based on basic health research data in 2018 from the ministry of health of the Republic of Indonesia, the prevalence of joint disease in Indonesia is around 7.3% of the total population of Indonesia [3]. One of the treatment options used to treat inflammatory osteoarthritis is ketoprofen [4]. 

Ketoprofen is a non-steroidal anti-inflammatory drug (NSAID) class that is effective for treating joint disorders [5]. Ketoprofen is commonly administered in oral dosage forms [4]. Nevertheless, the use of ketoprofen orally can cause bleeding in the gastrointestinal tract when the ketoprofen is not coated, or its release system is not modified [6]. In addition, the use of oral preparations can cause first-pass metabolism, so alternative routes of administration need to be considered [7]. Therefore, this drug has been developed in sublingual and parenteral dosage forms. The sublingual route may provide a convenient administration of ketoprofen; however, there is a possibility that it will be swallowed and there is a bitter taste to the drug [8]. Parenteral delivery can give higher bioavailability of the drug delivered, but this route may be painful, cause bleeding, need expert personnel to deliver, and produce needle waste disposal problems [9]. The transdermal route can be an alternative strategy because it does not undergo first-pass metabolism, is more convenient when compared to injection, does not require skilled personnel in its use, and reduces gastrointestinal side effects [5,7]. However, delivery via the transdermal route poses challenges because there is a barrier that must be passed, namely the *stratum corneum* [10].

One strategy to overcome the challenges of the transdermal delivery system is the use of microneedles. A microneedle is a micron-sized needle that is able to breach the *stratum corneum* without triggering nerve endings and blood capillaries so that it does not cause pain [11,12]. One type of microneedle is the dissolving microneedle (DMN) [13]. DMN has the advantage that the needle will dissolve, and the drug can be released into the skin after insertion [11]. However, the manufacturing of DMN depends on the water solubility of the loaded drug and the polymer matrix [14]. Therefore, DMN formulations require adequate drug solubility to mix with polymer to form a homogeneous matrix mixture [15]. Moreover, since ketoprofen is a BCS class II drug, it has low solubility, which may influence its permeation in the skin [5]. 

To overcome the problems documented above, the development of drugs by nanoparticles and co-grinding can be considered. Nanosuspension (NS) is a type of nanoparticle system that can be used to increase drug solubility. NS is a colloidal dispersion of submicron drug particles with a size < 1 µm [16]. Co-grinding is a method used to increase drug solubility by grinding solid drug compounds together with hydrophilic polymers [17]. Co-grinding is a simple and environmentally friendly technique because it does not use organic solvents [18]. Co-grinding has also been carried out for the drug ketoprofen, as previously performed by Hilaliyati [17]. They found that co-grinding ketoprofen with hydroxy propyl methyl cellulose can improve the physicochemical properties of ketoprofen and its dissolution rate in a phosphate buffer medium of pH 7.4. Therefore, this research explored the development of DMN formulation containing nanosuspension and CG ketoprofen using hydrosoluble polymers, such as poly(vinyl alcohol) (PVA) and poly(vinyl pyrrolidone) (PVP). A characterization and in vitro permeation study using Franz diffusion cells was also carried out. 

## 2. Results and Discussion

### 2.1. UV-Vis Spectrophotometric Condition

**Figure 1:**
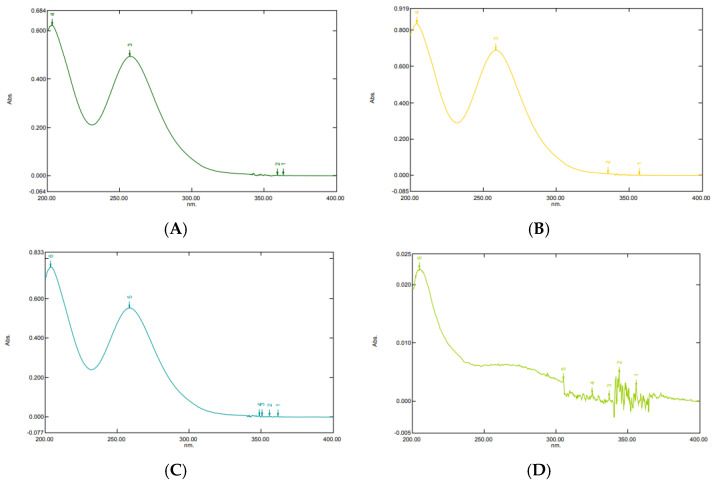
Absorption spectra of ketoprofen analyzed by spectrophotometer UV−Vis. (**A**) Ketoprofen standard. (**B**) Ketoprofen−PVA mixture. (**C**) Ketoprofen−PVP mixture. (**D**) Blank sample.

In this study, the analytical UV-Vis spectrophotometric condition refers to the previously optimized and validated method as per the International Harmonisation (ICH) guideline [19,20,21]. In this study, we performed the specificity and linearity assessments to verify the suitability of the spectrophotometric condition for analyzing and quantifying ketoprofen, and the protocol has been described in Section Validation of the UV-Vis Spectrophotometer. Figure 1A shows that a well-defined peak of ketoprofen was observed at 257 nm. Figure 1B,C reveals that the mixture of ketoprofen and polymers (PVA and PVP, respectively) does not interfere with the peak of ketoprofen. In addition, the absorption of blank sample (PVA solution without ketoprofen) in Figure 1D showed no peak at the specific wavelength of ketoprofen, thereby indicating the specificity of the method. The linearity assessment method was performed by analyzing pure ketoprofen across the calibration concentration range. The constructed calibration curve was found to be linear over the calibration range (2–12 µg/mL) with a correlation factor (R) of 0.9991 and linear regression equation y = 0.0567x + 0.1295. This result demonstrated a linear correlation existed between the absorbance and concentration of ketoprofen. Therefore, this UV-Vis spectrophotometric condition was determined to be suitable for the quantification of ketoprofen.

### 2.2. High-Performance Liquid Chromatography (HPLC) Validation Method

**Figure 2:**
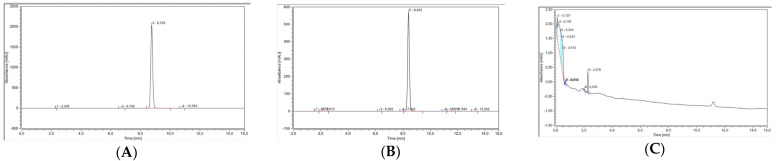
Chromatogram and spectrum of ketoprofen as determined by HPLC. (**A**) Ketoprofen standard. (**B**) Ketoprofen−PVA mixture. (**C**) Blank sample.

The chromatographic condition explained in Section Chromatographic Condition referred to previous optimized and validated method, as per the Food and Drug Administration (FDA) and European Medicine Agency (EMEA) bioanalytical method validation guidelines [22,23,24]. However, in this study, the method was partially revalidated according to the guidelines. The parameters measured were specificity, linearity, limit of detection (LOD), limit of quantification (LOQ), precision, and accuracy. The validation protocol has been explained in Section Validation of High-Performance Liquid Chromatography (HPLC) Method. Specificity was evaluated by confirming the ability of the analytical method to differentiate between the analyte and the other constituents in the sample matrix. As depicted in Figure 2A–C, the sample was separated efficiently, and there were no co-elution and matrix interference peaks found in the chromatograms of the ketoprofen-PVA mixture or blank solvent samples. 

The linearity assessment method was performed by analyzing pure ketoprofen within the calibration concentration range. The area of ketoprofen was linear over the calibration range (5–30 µg/mL) with a correlation factor (R) of 0.9997. As shown in Table 1, the LOD and LOQ for ketoprofen were 0.705 and 2.138 µg/mL, respectively. Therefore, this HPLC method was determined to be sensitive and suitable for the quantitative detection of ketoprofen. In quantitative analysis, accuracy and precision are the most important parameter validations. The accuracy and precision of this study were analyzed using recovery and relative standard deviation (RSD), respectively. The average recovery of each sample at different concentrations was in the range of 101.36–113.57%. In addition, the precision of each sample at different concentrations was 0.31–4.54%. The method validation guidelines presented by the ICH and US FDA allow for accuracy and precision in the range of 80–120% and less than 5%, respectively [20,24]. Therefore, this HPLC method provides excellent precision and accuracy at all concentrations in the sample matrix for analyzing ketoprofen.

### 2.3. Characterization of Nanosuspension Ketoprofen

#### 2.3.1. Particle Size and Zeta Potential Analysis

Each sample of ketoprofen NS with various concentrations of stabilizer was examined for particle size distribution and polydispersity index (PDI) using a Particle Size Analyzer (Malvern, UK), referring to the method Section Particle Size and Zeta Potential Analysis. The measurement results can be seen in Table 2. The results showed that ketoprofen nanosuspension formulation with 2% PVA (NS-3) produced a smaller particle size and PDI compared to NS-1 and NS-2. All NSs had particle sizes below 1000 nm. Measurement of the Dv-90 of NS formulations found that they had a particle size of less than 600 nm [25]. The PDI of NS-1 and NS-2 showed no significant difference with *p* value of 0.4596. On the other hand, the PDI of NS-3 compared to NS-1 and NS-2 showed significant differences with *p* values of 0.023 and 0.0497, respectively. These results indicated that the higher the stabilizer concentration, the smaller the particle size obtained. The presence of a stabilizer polymer plays an important role in particle size reduction by minimizing the crystallization or particle coalescence [26]. 

Based on Table 1, by increasing the amount of PVA, the zeta potential of the formulations decreased, but there is no significant difference among the formula tested (*p* value > 0.05). This may be attributed to the adsorption of nonionic polymers on the surface of solid particles leading to a decrease in charge of the diffusion layer [27].

**Figure 3:**
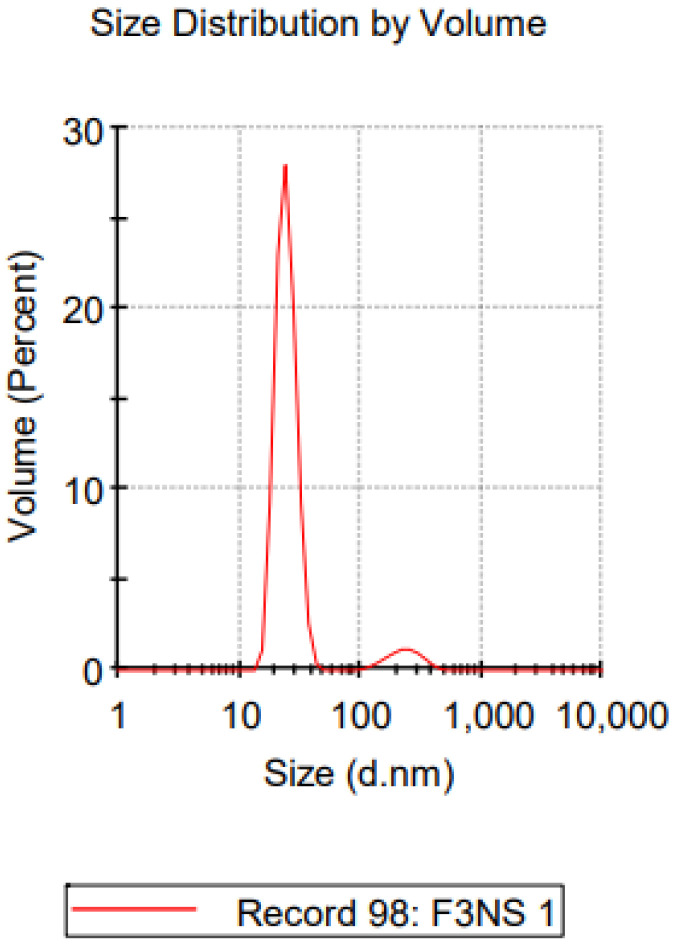
Representative of DLS graph of NS-3.

The representative dynamic light scattering (DLS) graph of particle size distribution analysis for NS-3 can be seen in Figure 3. The graph described that there are two peaks of particle size distribution that represent two populations of the particle distribution. However, the higher peak represents 93.3% of the nanosuspension volume with particle size of 24.92 nm, and the lower peak represents 6.7% of the nanosuspension volume with particle size of 237.1 nm. Therefore, the particle size distribution analysis showed that the nanosuspension is relatively dispersed, with >90% of the volume having a similar distribution size.

#### 2.3.2. Determination of Ketoprofen Content in Nanosuspension

The determination of the concentration of ketoprofen in the NS formulation was determined using a UV-Vis spectrophotometer in a phosphate buffer with a pH of 7.4, referring to the method Section Determination of Ketoprofen Content in Nanosuspension. The assay results obtained from NS-1 (PVA 0.5%), NS-2 (PVA 1%), and NS-3 (PVA 2%) were 87.15% ± 1.81, 91.56% ± 7.15, and 102.96% ± 0.26, respectively.

### 2.4. Characterization of Co-grinded Ketoprofen

#### 2.4.1. FT-IR Spectrophotometer

**Figure 4:**
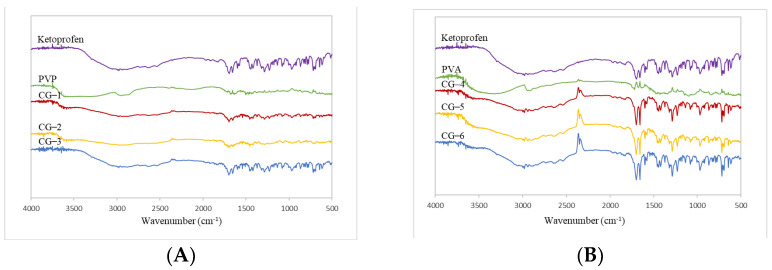
(**A**) Co−grinded Ketoprofen FTIR Spectrum with PVP and (**B**) Co−grinded Ketoprofen FTIR Spectrum with PVA.

FTIR spectrophotometer analysis was carried out to determine the possible interaction between ketoprofen and PVP and PVA polymers used in the manufacturing of co-grinding, referring to method Section FT-IR Spectrometer. The formulation for co-grinding ketoprofen can be seen in Table 3. Figure 4 shows the results of the FTIR spectrophotometer. 

Based on the data depicted in Figure 4A, there was no spectrum change in CG-1, CG-2, and CG-3 when compared to pure ketoprofen and PVP. On the other hand, the FTIR spectrum between CG ketoprofen and PVA exhibited a slight change. It can be seen from Figure 4B that there are wavenumbers at 2348 cm^−1^, which indicates that there was noise from the CO_2_ generated due to environmental condition changes [28]. The results revealed that there was no chemical interaction in the production of CG of ketoprofen with PVP or PVA.

#### 2.4.2. XRD

**Figure 5:**
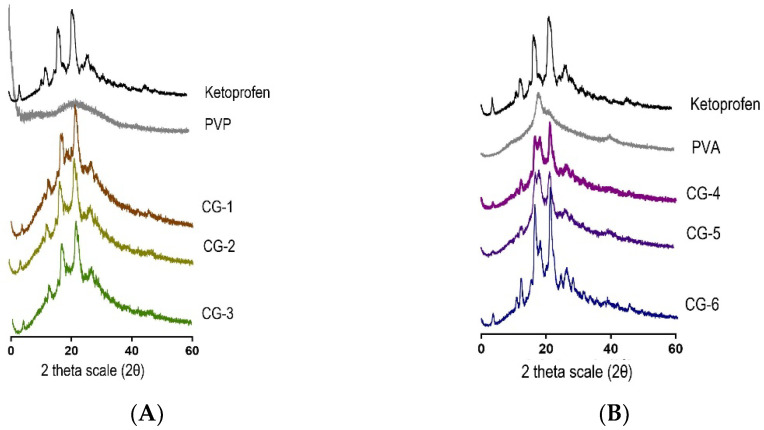
(**A**) Co-grinded Ketoprofen Diffractogram with PVP; (**B**) Co-grinded Ketoprofen Diffractogram with PVA.

X-ray diffraction analysis aimed to find the crystallinity of all the samples that have been made [29], referring to method Section XRD (X-ray diffraction). The diffractograms described in Figure 5 showed the diffraction characteristic of ketoprofen in the 13° to 28° region. These sharp peaks indicated that ketoprofen is in a crystalline form [30]. Figure 5A describes scattered peaks, which show that PVP has an amorphous nature but not PVA. Figure 5B depicts a sharp peak at 19.9°; thus, PVA has semi-crystalline properties [31,32]. XRD results for both CG of ketoprofen with PVP and PVA showed a sharp peak, which means that the CG product was in a crystalline state [33]. However, the intensity of the resulting Bragg peak is lower when compared to PVA, which has a higher molecular weight. PVP with lower molecular weight has a number of higher terminal groups, is less sterically obstructed and thus freer to interact with the carboxylic acid group of ketoprofen, and can cause interference with crystal formation [34].

#### 2.4.3. Differential Scanning Calorimetry (DSC) Analysis of Co-grinded Ketoprofen

**Figure 6:**
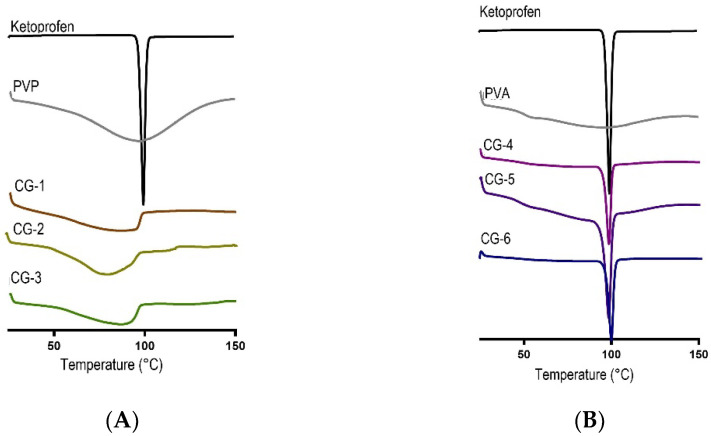
(**A**) Thermogram DSC Co-grinded Ketoprofen with PVP; (**B**) Thermogram DSC Co-grinded Ketoprofen with PVA.

This study aimed to find changes in the thermodynamic properties after the co-grinding process. This method has been described in Section Differential Scanning Calorimetry (DSC) Analysis of Co-grinded Ketoprofen. The properties can be seen when the material is subjected to heat [17]. Figure 6A,B display an endothermic peak of ketoprofen at 95.8 °C, which is associated with the melting point of ketoprofen. The sharp and narrow area under the curve indicated that ketoprofen was in a crystalline state. The pure PVP showed a wide endothermic peak at 94.5 °C, which explains the glass transition temperature (Tg) of this polymer due to its amorphous properties. In Figure 6B, there was a glass transition of the PVA thermogram at 48.8 °C, and an endothermic peak can be observed at 92.6 °C due to its semi-crystalline nature. No melting peak was produced at CG-1, CG-2, or CG-3. These showed that there were no traces of crystals in the co-grinding of ketoprofen and PVP. Co-grinding of ketoprofen and PVP gave a physical interaction that made the solid crystalline form of ketoprofen change to an amorphous phase [31]. Figure 6B also shows the endothermic peaks at CG-4, CG-5, and CG-6, which corresponds to the lower melting point of ketoprofen [30]. These results showed that there were traces of crystals in the co-grinding of ketoprofen and PVA. 

#### 2.4.4. Scanning Electron Microscopy (SEM)

**Figure 7:**
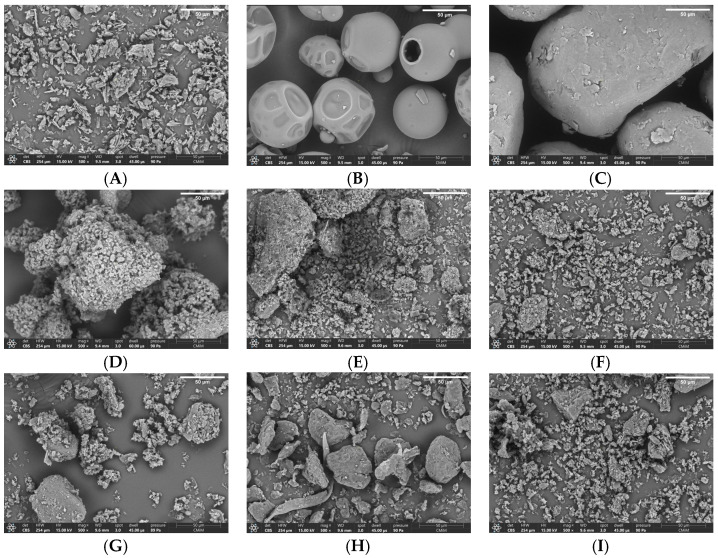
SEM Observations for (**A**) Ketoprofen, (**B**) PVP, (**C**) PVA, (**D**) CG-1, (**E**) CG-2, (**F**) CG-3, (**G**) CG-4, (**H**) CG-5, and (**I**) CG-6 at magnification of 500× (scale bar shown in the figure: 50 μm).

SEM was conducted to find the polymorphism and morphology of the prepared CG ketoprofen, referring to method Section SEM (Scanning Electron Microscopy). Based on the results shown in Figure 7A, it can be seen that ketoprofen was a crystalline solid with large irregular lumps and a hollow texture [17]. The pure powder of PVP (Figure 7B) produced a ball-shaped morphology with an uneven surface, as previously described [35]. In terms of PVA, as displayed in Figure 7C, the shape was like a large irregular lump. In the results of the co-grinding of ketoprofen and PVP (Figure 7D–F), the morphological form of ketoprofen is no longer visible because it has been evenly dispersed on the surface of the PVP. Similarly, the results of the SEM co-grinding of ketoprofen and PVA (Figure 7G–I) show that ketoprofen was homogeneously dispersed on a PVA hydrophilic polymer carrier. The results of the co-grinding formed a homogeneous one-phase system that cannot be distinguished as ketoprofen and PVP or ketoprofen and PVA. The average particle size was measured based on the SEM images and analyzed by using ImageJ for ketoprofen. The average particle sizes of PVP, PVA, CG-1, CG-2, CG-3, CG-4, CG-5, and CG-6, respectively, were 34.12 ± 2.47 µm, 69.19 ± 5.43 µm, 113.65 ± 9.97 µm, 55.59 ± 4.61 µm, 49.27 ± 2.40 µm, 43.18 ± 4.76 µm, 52.65 ± 6.05 µm, 49.88 ± 4.66 µm, and 38.41 ± 2.39 µm. 

### 2.5. Dissolution Study of Nanosuspension and Co-grinded Ketoprofen

**Figure 8:**
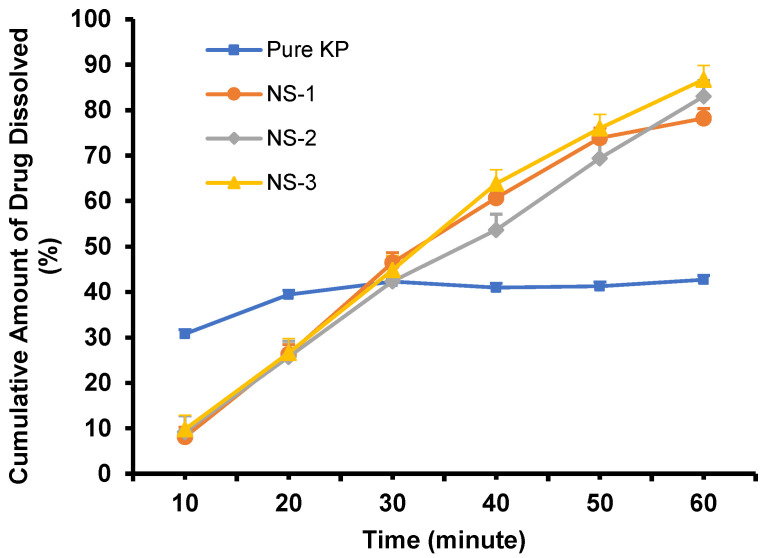
Dissolution profile curve of ketoprofen nanosuspension (n = 3, mean ± SD, *p* > 0.05).

The dissolution test aims to obtain a proof of concept that ketoprofen made in the NS and CG form can be dissolved in the dissolution medium (phosphate-buffered saline, pH 7.4), referring to method Section 3.2.6. The dissolution study was carried out on 5 mL of NS containing ~50 mg of ketoprofen and on 500 mg of the CG mixture containing ~166.67 mg of ketoprofen. The dissolution profile of pure ketoprofen, as depicted in Figure 8, showed less than 50% of the cumulative amount of the drug dissolved in the dissolution medium, and fluctuations occurred at 30, 40, and 50 min with insignificant differences among the time interval (*p* value > 0.05). As for the ketoprofen NS, the dissolution profile obtained showed that NS-3, the formulation with the highest concentration of PVA (2%), showed a higher amount of the drug that dissolved in the medium solution than NS-1 (PVA 0.5%) and NS-2 (PVA 1%). The results of the cumulative percentage of drug dissolution after 60 min for pure ketoprofen (Pure KP), NS-1, NS-2, and NS-3, respectively, were 42.69 ± 0.937, 78.20 ± 2.144%, 83.04 ± 3.466%, and 86.73 ± 3.094%. All ketoprofen-loaded NS samples had a higher cumulative drug amount dissolved when compared to pure ketoprofen. As the representative, the cumulative amount of drug dissolved of NS-3 compared to pure ketoprofen showed a significant difference with *p* value of 0.1618. This result showed that NS was able to enhance ketoprofen solubility in the medium used. The cumulative amount of drug dissolution increased as the concentration of PVA in the NS formulation increased. The increase in the cumulative amount of the drug may be attributed to the reduction in particle size, the increase in the surface area of the drug, and the increase in saturation solubility due to the presence of hydrophilic polymers [36]. 

As for the CG ketoprofen, the results of the X-ray diffraction test showed that the co-grinding of ketoprofen and PVA was a crystalline solid, although the largest increase in the dissolution profile should occur in the amorphous solid dispersion. Therefore, the crystallinity of the solid dispersion was not an obstacle to the dissolution process of the ketoprofen. Generally, amorphous solids have a higher solubility than crystals. However, amorphous solids can undergo a solution-mediated phase transformation to a metastable or crystalline form that is less soluble in the dynamics of the dissolution medium [37]. After the transformation phase, the less soluble solid will then cause a slower rate of dissolution. In this study, the partial crystallinity of CG-5 can increase the dissolution rate despite the presence of crystal traces [30].

In addition, because of the movement of the dissolution medium during the test, the hydrophobic domain, i.e., the drug, can crystallize or agglomerate during the process of release, which can lead to a slower dissolution rate. In the dissolution process of CG ketoprofen and PVP, agglomeration occurred. The increase in particle size reduces the effective surface area for solubilization during the dissolution process so that there can be a decrease in the dissolution rate [30]. In contrast, even though PVA was a semi-crystalline polymer with limited interaction with the ketoprofen, its excessive viscosity in solution may present a barrier for the solution, which mediates the transformation of the drug domain during dissolution. The PVA combination also showed that there was no agglomeration during the dissolution process [30]. Therefore, it can be concluded that the optimal formulation was CG ketoprofen: PVA = 1:2 (CG-5). 

**Figure 9:**
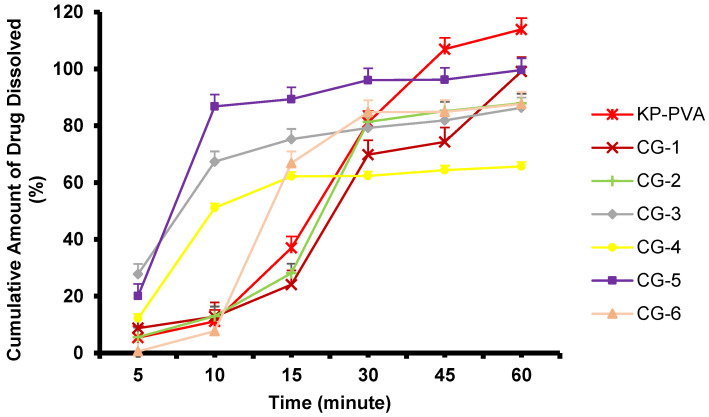
Dissolution profile curve of Co-grinded ketoprofen (n = 3, mean + SD, *p* > 0.05).

As can be seen in Figure 8 and Figure 9, the dissolution profile of pure ketoprofen showed that less than 50% of the cumulative amount of the drug dissolved in the dissolution medium after 60 min, while for the NS and CG formula, more than 80% of the cumulative amount of the drug dissolved after 60 min; this result shows that the NS method may enhance the solubility of ketoprofen.

Based on Figure 9, it can be seen that there was a difference in dissolution profile between the physical mixture of ketoprofen and PVA (KP-PVA) with CG-5. CG-5 can increase the dissolution rate, with more than 80% of the dissolved drug reached at 10 min, while the physical mixture only reached ~11%, showing that co-grinding may enhance the dissolution rate of ketoprofen. When compared with pure ketoprofen, the addition of PVA can increase the dissolution rate. However, the physical mixture of ketoprofen and PVA demonstrated a quicker dissolution rate when compared to the ketoprofen nanosuspension formula. This is related to the release strategy of ketoprofen in the form of nanoparticles as a controlled release system, which is discussed further in Section 2.6.9.

### 2.6. Evaluation of a Dissolving Microneedle (DMN) Containing Nanosuspension and Co-grinded Ketoprofen

#### 2.6.1. Physical Evaluation of Dissolving Microneedle 

The physical evaluation method refers to the method Section Physical Evaluation of Microneedle Array. The physical evaluation of DMN containing ketoprofen NS produced from the formulations with combined polymers produced a sturdier structure than that manufactured using a single polymer. A similar study conducted by Permana et al. [38] also showed that microneedles with single polymers had poorer mechanical strength compared to the combined one. The morphological study depicted in Table 4 showed that all formulations had a flat baseplate, sharp needles, and no deposit on the tip of the needle. Similar to the DMN containing NS, the dissolving microneedle containing CG ketoprofen also showed that the single polymer formulation (F1-MN CG and F2-MN CG) had poor mechanical strength; thus, the formulations prepared using polymer combination were selected. The formulations were F6-MN CG, F7-MN CG, F8-MN CG, F9-MN CG, F10-MN CG, and F11-MN CG. These formulations did not produce any precipitation, and the needles formed were not brittle when removed from the mold. However, F3-MN CG, F4-MN CG, and F5-MN CG, fabricated using polymer combinations, also produce brittle needles. This may be caused by the high rate of evaporation that occurs during the drying process [39]. The results of the morphological study of dissolving microneedle loaded with Co-grinded ketoprofen can be seen in Table 5. 

#### 2.6.2. Mechanical Properties

Mechanical strength evaluation was carried out to ensure the strength of the microneedle against the applied pressure, with reference to the method section Mechanical Properties. The results are listed in Table 6 F1-MN NS, manufactured using PVA, showed a height reduction of 13%, which also indicated that the single polymer showed the poor mechanical property. F2-MN NS, which contained PVP as a single polymer, showed the highest percentage of needle height reduction in the needle count (>18%), representing poor mechanical ability. Based on previous research, it has also been found that films made from PVP alone have poor mechanical strength [38]. According to Teodorescu et al. [40], PVP produced a hygroscopic and brittle property. In order to improve the mechanical strength of PVP, different hydrophilic polymers can be added. Combining PVP with PVA can be used to increase the mechanical strength of DMN. F4-MN NS and F5-MN NS with a combination of polymers showed the lowest percentage of needle height reduction. The combination of PVA and PVP has the possibility of increasing the formulation ability due to the interaction between the OH group in PVA and the C=O group in PVP [38]. 

Similarly, dissolving microneedles containing CG ketoprofen using a polymer combination produced promising mechanical strength, as can be seen in Table 6. F9-MN CG exhibited poor mechanical strength due to the highest reduction in needle height. Increasing the concentration of PVP caused a significant increase in the mechanical strength of the microneedle (*p* < 0.05), which was indicated by the decrease in needle height measured after the test. Therefore, the addition of PVP concentration in the formulation affected the mechanical strength of the microneedle. In addition, the lower the addition of PVA concentration, the worse the mechanical properties of DMN are [41]. This is because PVA is a hygroscopic polymer and also has a low flexural modulus, which causes it to be flexible and unable to withstand the applied pressure [42].

#### 2.6.3. Loss of Mass

The loss of mass during the drying process was calculated to find the percentage of mass loss by weighing the gel solution before and after drying the dissolving microneedle, referring to the method section Loss of Mass while Drying. The percentage loss of mass of the four microneedle formulations is in the range of 54–92%, as shown in Table 7.

The results displayed in Table 7 show that the higher the PVP concentration in the DMN formulation, the lower the loss of mass is. This can be caused by PVP, which can reduce the evaporation of water from the wet mass of the DMN gel blend. In addition, PVP is also hygroscopic, so it may attract water from the surrounding environment. Loss of mass indicates the amount of water evaporated from the DMN gel mass. The reduced water content due to evaporation affects the drug concentration in the DMN but does not affect the amount of the drug since only water is what evaporates. The drug concentration changes as the total mass of the DMN becomes smaller due to mass loss.

#### 2.6.4. Insertion Ability Study

**Figure 10:**
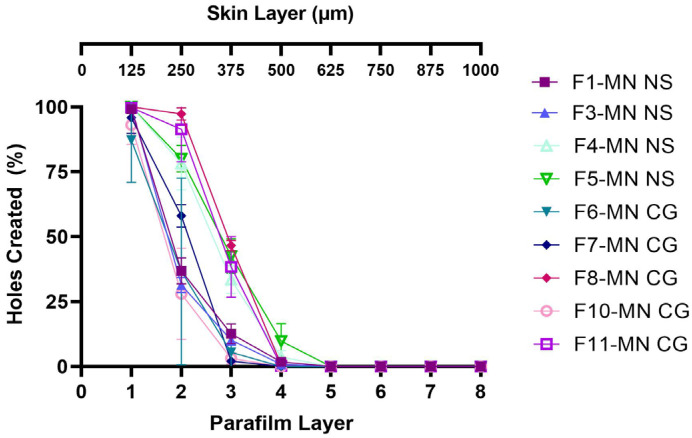
Illustration of the percentage of holes created in Parafilm^®^ M after DMN containing nanosuspension and Co-grinded ketoprofen insertion (n = 3, mean ± SD, *p* > 0.05).

According to Larrañeta et al. [43], DMN’s ability to penetrate the skin is the main prerequisite ability before DMN begins to dissolve and deliver drugs into the skin. The insertion ability study refers to the method section Insertion Ability Study. The insertion ability test was carried out by applying DMN to the Parafilm^®^ M as a skin simulation. Each Parafilm^®^ M layer has a thickness of 126 ± 7 µm [44]. When the tested DMN can reach the fourth layer of Parafilm^®^ M, this shows that DMN may penetrate the skin to a depth of ~500 µm. If it has sufficient mechanical properties, DMN can penetrate the Parafilm^®^ M layer to deeper layers. The thickness of the stratum corneum and epidermis of human skin is 10–15 µm and 50–100 µm, respectively. DMN’s ability to penetrate Parafilm^®^ M is related to its mechanical strength upon insertion into the skin as well as the resulting depth of insertion in the skin [45]. Based on the data presented in Figure 10, F4-MN NS (PVA 5%-PVP 10%) and F5-MN NS (PVA 5%-PVP 15%) penetrated the third to the fourth layer of Parafilm^®^ M, indicating that the maximum distance reached by the two DMN formulations was 375–500 µm. Nevertheless, F1-MN NS (PVA 10%) and F3-MN NS (PVA 5%-PVP 5%) could only penetrate the third layer of Parafilm^®^ M with a percentage of 9.78% and 12.45%, with few holes created in the fourth layer. These results also showed that F4-MN NS and F5-MN NS had better insertion capabilities than F1-MN NS and F3-MN NS. This is in line with the mechanical property test where F4-MN NS and F5-MN NS proved to be stronger mechanically so that they could penetrate Parafilm^®^ M to a deeper layer. Thus, it was determined that F4-MN NS and F5-MN NS were more suitable formulations for further evaluations.

For DMNs containing CG ketoprofen, the test results can also be seen in Figure 10. F8-MN CG and F11-MN CG can penetrate the third layer, which created a number of holes of more than 25%, and this was different from other formulations that only produced less than 10% of holes created in the third layer. In addition, F8-MN CG and F11-MN CG were also able to penetrate the fourth layer of Parafilm^®^ M. However, there were no significant differences between all of these data (*p* > 0.05). This was in accordance with the mechanical strength produced by F8-MN CG. This formulation showed the greatest mechanical strength among the other formulations tested. Based on the results, it was found that F6-MN CG, F7-MN CG, and F10-MN CG can penetrate up to 375 μm, while F8-MN CG and F11-MN CG can penetrate up to 500 μm. Thus, F8-MN CG and F11-MN CG were also selected for the next steps.

#### 2.6.5. In-Skin Dissolution Study

**Figure 11:**
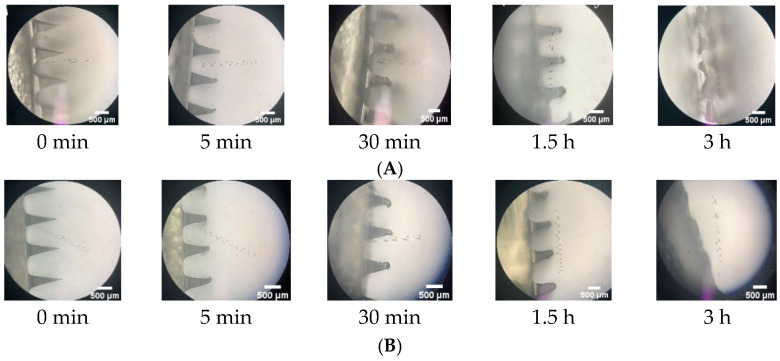
Microscopy observation for in-skin dissolution studies containing ketoprofen nanosuspension in DMN formulations (**A**) F4-MN NS (5% PVA + 10% PVP) and (**B**) F5-MN NS (5% PVA + 15% PVP) (scale bar: 500 μm).

The formulations tested in this study were F4-MN NS, F5-MN NS, F8-MN CG, and F11-MN CG. An in-skin dissolution study was performed by inserting DMN in full-thickness rat skin, referring to the method section In-Skin Dissolution Study. DMN applied to the rat skin was observed at certain time intervals periodically under a digital microscope to see the solubility of the DMN array needle. Based on Figure 11, F4-MN NS completely dissolved after 3 h, and F5-MN NS completely dissolved after 3.5 h. Based on the components of each DMN, F5-MN NS contained a higher PVP concentration than F4-MN NS (15%), while F4-MN NS contained 10% PVP with the same PVA concentration in both formulations. This indicated that PVP as a polymer plays an important role in the DMN dissolution process. A higher concentration of PVP has the potential to cause an increase in the stiffness and density of DMN, so it takes a longer time for complete dissolution [45].

**Figure 12:**
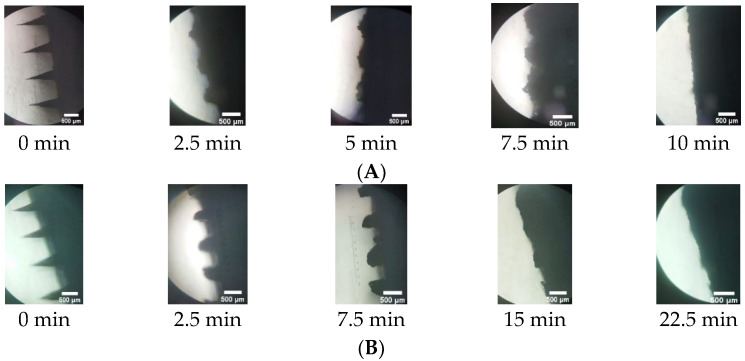
Microscopy observation for in-skin dissolution studies containing Co-grinded ketoprofen in DMN formulations (**A**) F8-MN CG (5% PVA + 15% PVP) and (**B**) F11-MN CG (7.5% PVA + 15% PVP) (scale bar: 500 μm).

Based on Figure 12, the results showed that the F8-MN CG needle part completely dissolved after 10 min, while the F11-MN CG completely dissolved after 22.5 min. This showed that F8 has a faster dissolution time than F11. PVP and PVA are polymers that can dissolve well in body fluids. This is due to the hygroscopicity and water absorption properties of PVP and PVA. When PVP and PVA are applied to the skin, PVP and PVA will immediately absorb the surrounding interstitial fluid and then dissolve [46]. However, PVA, which is semicrystalline, will dissolve more slowly than PVP [44]. Therefore, F11-MN CG containing a higher concentration of PVA dissolved more slowly than F8-MN CG. The results also showed that DMN NS gave a longer dissolution time when compared to DMN CG. This might be caused by the use of PVA as a stabilizer for manufacturing the NS formulation. The use of PVA in the NS may increase the density of the DMN prepared, resulting in a longer dissolution process.

#### 2.6.6. Morphological Observation of Selected Formulation

**Figure 13:**
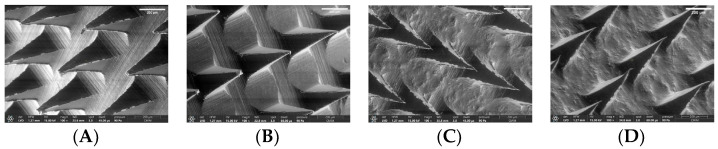
SEM Observation Results for the Selected Formulation (**A**) F4-MN NS, (**B**) F5-MN NS, (**C**) F8-MN CG, and (**D**) F11-MN CG (scale bar: 200 μm).

Based on Figure 13, it can be seen that the analysis of the DMN formulation containing CG ketoprofen, namely F8-MN CG and F11-MN CG, had a rough surface compared to the DMN formulation containing ketoprofen NS, namely F4-MN NS and F5-MN NS. On the other hand, F4-MN NS and F5-MN NS have smooth and homogeneous surfaces. 

#### 2.6.7. Determination of Ketoprofen Content

Determination of ketoprofen levels in DMN needles was carried out at days 0 and 30 after the drying process to see the possibility of degradation or decrease in ketoprofen content in DMN during storage, referring to method section Determination of Ketoprofen Content in Dissolving Microneedles. After 30 days, there was a decrease in ketoprofen levels in DMN needles containing ketoprofen NS. F4-MN NS (PVA 5%-PVP 10%) decreased content by 35.82%, while F5-MN NS (PVA 5%-PVP 15%) decreased content by 41.64%. In DMN containing CG ketoprofen, there was a decrease in ketoprofen levels after being stored for 44 days. F8-MN CG (5% PVA—15% PVP) decreased content by 39.39%, and F11-MN CG (7.5% PVA—15% PVP) decreased content by 29.00%. During the storage period, DMN was stored at room temperature in a closed transparent plastic box. The degradation of the drug is an important concern because it can lead to reduced therapeutic activity. Degradation of ketoprofen may be caused by not storing the DMN prepared in a light-proof container. This potentially caused photolysis of ketoprofen [47]. Ketoprofen is a 2-propionic acid derivative of benzophenone. The aryl part of this drug molecule contains many chromophore groups, which have significant absorption in the UV-A and UV-B regions. This chromophore includes the benzophenone part of ketoprofen. The ionized form of ketoprofen can undergo rapid decarboxylation on photolysis [47].

#### 2.6.8. Differential Scanning Calorimetry (DSC) Analysis of DMN

**Figure 14:**
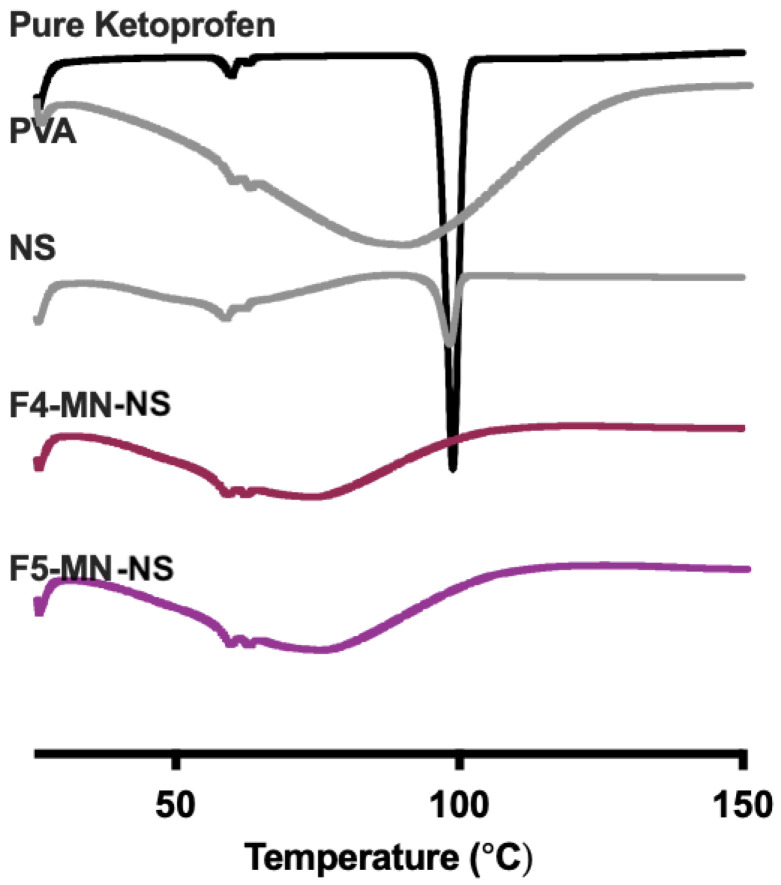
Thermogram DSC of ketoprofen-loaded NS and MN.

In this research, the DMNs were also made of the polymer and NS. Therefore, it is important to investigate the physical stability of the NS and DMN-containing ketoprofen-loaded NS. This method refers to method Section 3.2.8. Figure 14 describes the thermal stability of pure ketoprofen, PVA (NS material), ketoprofen-loaded NS, and selected MN containing NS. Based on the results, it is known that in the NS thermogram, the sharp endothermic peak of ketoprofen was still found with a lower intensity when compared to the pure drug at 100 °C. Formulating ketoprofen into NS could enhance its solubility, but crystal traces of this drug were still observed. Nevertheless, this sharp peak was not seen in either F4-MN-NS or F5-MN-NS. These showed that there were no traces of crystals in these MN formulations because the physical interaction among the components used may alter the solid crystalline form of ketoprofen changed to an amorphous phase [25,48].

#### 2.6.9. In Vitro Permeation Studies

**Figure 15:**
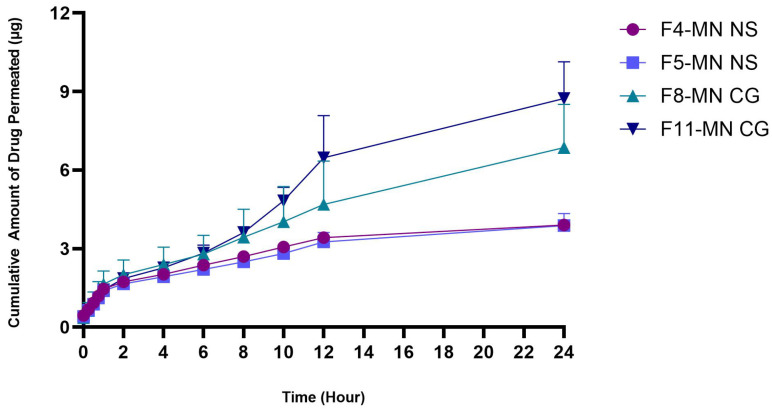
The cumulative amount of drug permeated in vitro using Franz diffusion cells for 24 h from DMN loaded with nanosuspension and Co-grinded ketoprofen (n = 3, mean ± SD, *p* > 0.05).

An in vitro permeation study in PBS at pH 7.4 was performed to determine the amount of the drug that can permeate into the receptor compartment. This study was carried out using a Franz diffusion cell. This cell diffusion simulates the transdermal delivery of drugs applied to the skin [49]. Permeation studies can be performed with a Franz diffusion cell system using a membrane placed between the donor compartment and the receptor compartment, referring to method Section 3.2.9. For this study, synthetic membranes such as polysulfone and biological membranes such as animal skins can be used [50]. In this study, the skin membranes of female white rats of the Sprague Dawley strain were used. The medium used was a phosphate-buffered solution (PBS) of pH 7.4. The in vitro permeation study was carried out on F4-MN NS and F5-MN NS, each containing ~50 mg of ketoprofen, and also on F8-MN CG and F11-MN CG, each containing ~166,67 mg of ketoprofen. After conducting the permeation test for 24 h with 12 sampling points, the cumulative amount of drug permeated into the receptor compartment from F4-MN NS and F5-MN NS was 3.90 ± 0.07 µg and 3.88 ± 0.46 µg, respectively. Based on Figure 15, F4-MN has more drug permeated when compared to F5-MN NS. F4-MN NS was a formulation containing a polymer combination of 5% PVA and 10% PVP, while F5-MN NS contains a polymer combination of 5% PVA and 15% PVP. However, the amount of ketoprofen permeated from the two DMN formulations was not significantly different, with a *p*-value = 0.7761. The average amount of drug contained in the DMN needle section was ~30 µg, but only about ~3.8 µg permeated into the skin. The percentages of the drug that permeated into the compartment receptors after 24 h for F4-MN NS and F5-MN NS were 13.41 ± 2.46% and 12.26 ± 2.21%, respectively.

In terms of the CG system, the cumulative amount of the drug that permeated after 24 h of study for F8-MN CG and F11-MN CG was 6.86 ± 1.65 µg and 8.73 ± 1.40 µg, respectively. This result was not significant because the *p* value was higher than 0.05, namely 0.7552. In the needle section, DMN had an average of ~68 mg of ketoprofen, but only about ~7.8 µg was permeated into the skin. The percentage of the drug that permeated into the receptor compartment after 24 h for F8-MN CG and F11-MN CG was 9.37 ± 1.48% and 13.67 ± 1.56%, respectively. This percentage was obtained from the comparison of the cumulative amount of the drug that permeated after 24 h with the amount of drug contained in the DMN needle. These results indicated that more than 80% of the ketoprofen in the DMN needle had not been delivered to the receptor compartment within 24 h. However, all CG formulations showed a higher amount of cumulative drug permeated when compared to the NS formulations. For instance, the permeation profile of F11-MN CG produced a significant difference compared to F4-MN NS and F5-MN NS with a *p* value of 0.0061 and 0.0055, respectively. This result indicated that the CG ketoprofen formulation may increase the amount of drug permeated while the NS formulation tends to have a smaller amount of drug permeated. This difference in the amount of drug permeated between the MN-NS and MN-CG formulas may indicate the release of DMN containing nanosuspension as a controlled system which will be discussed further in this section.

The results of this study were in line with McGrath et al. [51], who examined DMN containing ketoprofen with the atomized spray method. In that study, the amount of the drug that permeated was about 5.6 ± 1.9 mg, or 27% of the total drug, after 24 h. In this study, less than 15% of the drug was permeated into the receptor compartment medium. These results were in accordance with the range obtained in other studies looking at microneedle-mediated drug permeation, where the cumulative amount of drug permeated ranged from 5% (insulin) to 16% (metronidazole) [52]. Based on the results of permeation tests that have been carried out, controlled-release delivery of ketoprofen NS via DMN may be considered. Ketoprofen is also a suitable model drug to be formulated in controlled-release preparations because it has a short plasma elimination half-life and poor solubility in water [53]. 

In a previous study by He et al. [54], they investigated a controlled-release drug delivery via DMN. In that study, etonogestrel-loaded DMN in the form of microcrystalline particles permeated as much as 23.34 ± 1.36% after the first 24 h and continued for 7 days. Another study conducted by Permana et al. [38] also investigated the controlled release of antifilarial DMN in the form of solid lipid nanoparticles for 48 h. Combining DMN with nano- or microparticle technology can be a strategy to regulate controlled drug release. The polymer used also affects drug release from DMN [55]. The polymers used in this study were PVA and PVP, where PVA is a polymer with a slow dissolution rate in an aqueous medium; therefore, the delivery system in this study may be suitable for a controlled-release system. A similar study was conducted by Tekko et al. [56], who investigated the release of DMN methotrexate for rheumatoid arthritis with PVA/PVP/HPMC polymers; the results showed that methotrexate release occurred for more than 24 h continuously. In this study, the permeation test for DMN ketoprofen was only carried out for 24 h, with the cumulative amount increasing at each sampling time interval. Further research is required to see the sustainable release of the drug. 

The low permeation of drugs through DMN can be caused by various aspects, such as drug concentration, membrane thickness, lipid content, type of membrane used, contact time, degree of skin hydration, and skin pretreatment [57]. Rat skin used as a membrane affects the amount of drug that can be permeated, namely the type of full-thickness skin (full thickness) or dermatomed skin. In this study, the skin used was full-thickness skin, which could reduce drug permeation when compared to dermatomed skin. This can cause the drug to be deposited on the full-thickness skin and not reach the receptor compartment. Ameri et al. [58] previously compared microarray-coated zolmitriptan delivery to full-thickness skin and dermatome skin. In their study, the total drug amount that permeated through the skin of the dermatome into the receptor compartment was 85%, while that permeated through the full-thickness skin was 53%. This proves that the skin type used in the permeation test can affect the amount of drug delivered. In full-thickness skin, the drug must diffuse through the intact dermis to the receptor compartment. On the other hand, the lower part of the dermis at the capillary bed in the dermatomed skin has been removed so that the drug can reach the receptor compartment more quickly compared to the full-thickness skin. Full-thickness skin with a dermis without blood flow will result in a rate-limiting step in drug absorption into the skin so that it can reduce the amount of drug permeated [58]. However, the permeation study conducted in this work showed that dissolving microneedles containing NS and CG ketoprofen could penetrate the skin and deliver the drug in vitro.

## 3. Materials and Methods

### 3.1. Materials

Ketoprofen (Mepro, Indonesia), poly(vinyl alcohol) (Sigma Aldrich, Singapore), poly(vinyl pyrrolidone) (BASF, Indonesia), distilled water (Brataco, Indonesia), buffer phosphate (Merck, Germany), acetonitrile (Merck, Germany), ethanol 96% (Merck, Germany), Parafilm M (Bemis Company Inc, Soignies, Belgium), aluminum foil (Klin Pak, Indonesia), and sodium hydroxide (Merck, Germany) were used. Other chemicals and reagents used in this research were of analytical grade.

### 3.2. Methods

#### 3.2.1. Analytical Method Condition

##### UV-Vis Spectrophotometric Condition

The concentration of ketoprofen in NS and CG was determined using a UV-Vis spectrophotometer (Shimadzu, Japan) with a phosphate buffer of pH 7.4 as the solvent and analyzed at a wavelength of 257 nm. The measured ketoprofen concentration was obtained from the linear regression equation of the calibration curve. The calibration curve was obtained in the concentration range of 2, 4, 6, 8, 10, and 12 µg/mL of standard ketoprofen [59]. The analytical spectrophotometric condition used in this study refers to the previously optimized and validated method as per the International Council for Harmonisation (ICH) guideline [19,20,21].

##### Validation of UV-Vis Spectrophotometer

The validation of a UV spectrophotometric analytical method was tested using several parameters, including specificity and linearity [20,21]. To evaluate the specificity of the method, the UV spectra of blank samples (PVA or PVP) were compared to a ketoprofen standard solution. The analysis of NS and CG ketoprofen was also performed at 257 nm. The linearity of the proposed method was confirmed by preparing three different standard solutions of ketoprofen (2, 4, 6, 8, 10, and 12 µg/mL) and analyzing them in triplicate to create nine derived analytical curves. The linearity was assessed based on the linear regression curve. 

##### Chromatographic Condition

Quantitative analysis of ketoprofen in this study was carried out using a high-performance liquid chromatography (HPLC) system (Thermo Fisher Scientific, Waltham, MA, USA). A C18 column (Sunfire^TM^, 5µm; 250 × 4.6 mm) was used for the separation. The mobile phase employed was a mixture of phosphate buffer (pH 3.5) and acetonitrile with a ratio of 50:50. Samples were injected at a volume of 20 μL with a flow rate of 1 mL/min and detected at a wavelength of 257 nm under an isocratic condition [22]. This chromatographic condition employed refers to a previously optimized and validated method, as per the European Medicine Agency (EMEA) bioanalytical method validation guidelines [23,60].

##### Validation of High-Performance Liquid Chromatography (HPLC) Method

The optimized RP-HPLC method was subjected to method validation. The validation process was performed based on the ICH Validation of Analytical Procedures Q2 Analytical Validation [20], which included the following parameters: specificity, linearity, limit of detection (LOD), limit of quantification (LOQ), accuracy, and precision.

- Specificity

Specificity is a parameter that ensures there are no signal interferences caused by impurities or degradants in the analytical sample of the drug. To test specificity, the solvent used for dissolving ketoprofen was injected (20 µL of blank PBS pH 3.5 and acetonitrile) followed by 20 µL of ketoprofen solution (10 µg/mL). This test was repeated at least three times, and each chromatogram of the sample was compared to that of the blank PBS pH 3.5 and acetonitrile. A blank PVA solution (1% *w*/*v*) in PBS was also prepared to determine specificity, and each solution was filtered using a 0.22 µm filter prior to the injection into the HPLC system.

b. Linearity

In the ICH Validation of Analytical Procedure: Q2 (R1), linearity, defined as the ability of the method to produce results that are proportionate to the analyte concentration within a given range, was evaluated using working standard solutions of ketoprofen with concentrations of 5, 10, 15, 20, 25, and 30 µg/mL. An aliquot of each concentration was injected into the HPLC system using the optimized method to construct a calibration curve, where the x-axis represents concentration, and the y-axis represents the area obtained from HPLC. The correlation coefficient (R) was then calculated.

c. Limit of Detection (LOD)

The limit of detection (LOD) is the lowest concentration of analyte in a sample that can be detected but not necessarily quantified accurately. LOD was determined by injecting known concentrations of analyte and using the equation below:

(1) $$\mathrm{LOD}=\frac{3.3\sigma }{S}$$

where is the standard deviation (SD) of the response of the data used for constructing the regression line, and S is the slope of the line.

d. Limit of Quantification (LOQ)

The limit of quantification (LOQ) is the lowest concentration of analyte in a sample that can be accurately and quantitatively measured. LOQ can also be calculated using the equation below.

(2) $$\mathrm{LOQ}=\frac{10\sigma }{S}$$

where is the SD of the response of the data used, and S is the slope of the constructed calibration curve.

e. Accuracy

Accuracy is the closeness between the accepted reference value and the obtained value. Ketoprofen standard solutions with low, medium and high concentrations (5, 15, and 30 µg/mL) were used in the evaluation. To evaluate accuracy, each solution was injected into the HPLC system using an optimized method. Following the assay, the percent recovery and %CV were calculated using the calibration curve constructed. Accuracy testing must be performed within one day and between at least three different days, with three samples in each test cycle. The percentage recovery should be in the range of 80–120%.

f. Precision

Precision in analytical methods refers to the ability to obtain similar measurements from a homogeneous sample under consistent conditions. To assess precision, ketoprofen standard solutions with concentrations of 5 µg/mL (low), 15 µg/mL (medium), and 30 µg/mL (high) were injected as 20 µL aliquots into the HPLC system. Percent recovery and %CV were then calculated. Precision testing must be conducted within a day and between at least three different days, with three samples in each test cycle. Precision is typically presented as the standard deviation (SD) or %RSD of the measurements obtained.

#### 3.2.2. Formulation of Ketoprofen Nanosuspension

Ketoprofen NS was prepared using ultrasound equipment (Sonica Laboratory, Bengaluru, India) for 5 min with a pulse of 10 s and a pulse of 5 s, and the amplitude was 80%. This method refers to Vora et al. [25]. Ketoprofen-loaded NS was prepared with PVA dispersion at a concentration of 0.5% *w*/*v*, 1% *w*/*v*, and 2% *w*/*v* in ultrapure water. Approximately 200 mg of ketoprofen was weighed and then added into the PVA solution while it was stirred using a magnetic stirrer until homogeneous. The homogeneous suspension of ketoprofen was then given ultrasonic energy with an ultrasound device to form an NS [59].

#### 3.2.3. Characterization of Nanosuspension Ketoprofen

##### Particle Size and Zeta Potential Analysis

Particle size analysis and zeta potential of each formulation were measured using a particle size analyzer and zetasizer. A drop of the NS ketoprofen was diluted in 10 mL of distilled water; then, 1 mL was taken and put into a cuvette, and the particle size distribution of the NS ketoprofen was obtained. A particle size analyzer can also be used to measure the polydispersity index and zeta potential [50].

##### Determination of Ketoprofen Content in Nanosuspension

Method for determination of ketoprofen content in nanosuspension referring to method (Section UV-Vis Spectrophotometric Condition).

##### Determination of Selected Nanosuspension Ketoprofen Formula

The optimum NS ketoprofen formulation was selected based on the characterization results and formulated to DMN. The most promising NS formulation was the one that gave the smallest particle size distribution and had a polydispersity < 0.8 [50]. 

#### 3.2.4. Formulation of Co-grinded Ketoprofen

CG ketoprofen was made by grinding together a certain amount of ketoprofen and PVA in a ratio of 1:1, 1:2, and 2:1, as well as ketoprofen and PVP in a ratio of 3:1, 5:1, and 10:1 using a mortar and pestle (±60 min), and the results obtained were sieved using a 120 mesh sieve [61]. After the synthesis of CG ketoprofen, further characterization was carried out.

#### 3.2.5. Characterization of Co-grinded Ketoprofen 

##### FT-IR Spectrometer

The infrared spectra of ketoprofen powder, PVA, PVP, and CG mixtures were prepared by dispersing the sample on compressed KBr pellets under high pressure. Furthermore, they were measured by the percent transmittance of the wave numbers 4000 cm^−1^ and 600 cm^−1^ using FTIR (Shimadzu, Kyoto, Japan) [62].

##### XRD (X-ray Diffraction)

The X-ray diffraction pattern was determined using a diffractometer (Bruker D8 Advance). The measurement conditions are as follows: Cu metal target, Kα filter, and X’celerator detector; the voltage is 40 kV, the current is 30 mA, and the analysis is carried out in the 2 theta (θ) range of 100–100° [63]. The sample was placed in a sample holder (glass) container and leveled to prevent particle orientation during sample preparation. This analysis showed the diffraction pattern of the single compound ketoprofen, PVA, PVP and the solid dispersion formed [64].

##### Differential Scanning Calorimetry (DSC) Analysis of Co-grinded Ketoprofen

Thermal analysis was carried out using a DSC (Perkin Elmer Pyris 6 DSC) device, which was fed with nitrogen at a speed of 20 mL/min. In a closed aluminum container, ±2 mg of the sample was placed and heated at a rate of 10 °C/min from a temperature of 50–200 °C [65].

##### SEM (Scanning Electron Microscopy)

Microscopic analysis was carried out using a Scanning Electron Microscopy (Hitachi SU-3500) tool by observing the morphology of the crystals. The sample powder was placed in an aluminum sample container and coated with gold with a thickness of ±20 nm. Samples were observed with various magnifications on SEM. We set the voltage at 15–20 kV and current 12 mA [63].

#### 3.2.6. Dissolution Study

The dissolution study of NS ketoprofen was carried out referring to Iskandar [59] with modifications. The dissolution test was conducted at 37 °C ± 0.5 °C at 100 rpm. The medium used was phosphate-buffered solution of pH 7.4 in as much as 100 mL. The sample solution was taken at 10, 20, 30, 40, 50, and 60 min, taking 5 mL of solution, and then analyzed by a UV-Vis Spectrophotometer. The determination of the dissolution profile of CG samples was carried out using the sample in the form of a solid dispersion that was inserted into a hard gelatin capsule. The dissolution test used a type I dissolution apparatus (Electrolab TDT-08L, Bombay, India) with a speed of 100 rpm, the dissolution medium was 500 mL of phosphate-buffered solution at pH 7.4, and the temperature was set at 37 °C ± 0.5 °C. A total of 5 mL of sample solution was pipetted at 5, 10, 15, 30, 45, and 60 min, and upon each pipetting, the dissolution medium was replaced with a new one with the same volume and temperature when pipetting. Next, the sample solution was analyzed by a UV-Vis spectrophotometer at its maximum absorption wavelength [17]. The analytical spectrophotometric condition was used referring to the method section UV-Vis Spectrophotometric Condition.

#### 3.2.7. Formulation and Evaluation of Dissolving Microneedle (DMN) Containing Ketoprofen Nanosuspension or Co-Grinding

Ketoprofen in the form of NS and CG optimization results are contained in DMN. DMN was prepared by the micromolding method. The gel solution was obtained from a mixture of NS or CG containing ketoprofen added to the polymer solution, cast on a silicone micromold (15 × 15 needle holes, each measuring a height of 600 μm, base width of 200 μm, and an interspacing of 50 μm) and then put into a positive air pressurized chamber (AirPro, Taipei, Taiwan) at a pressure of 3–4 bar for 15 min, then dried for 48 h at a temperature of 30 °C [57,66].

##### Physical Evaluation of Microneedle Array

Surface morphology of DMN containing ketoprofen-loaded NS or CG samples was observed using a digital microscope and SEM [25].

##### Mechanical Properties 

The mechanical properties of DMN were evaluated using the TA-XT2 Texture Analyzer in compression mode. The height of the microneedle before compression was measured using a digital microscope. The microneedle array was then attached using double-sided adhesive tape to the cylindrical probe of the texture analyzer and moved downward facing a stack of Parafilm^®^ M. The texture analyzer runs at a rate of 0.5 mm/s for 30 s with a force of 32 N. Post-compression microneedle height was measured again using a digital microscope [66,67]. The percentage of needle height reduction was calculated using the equation below:

(3) $$\%\;\mathrm{Height}\;\mathrm{reduction}=\frac{Hb-Ha}{Hb}\;\times \;100\%\;$$

where *Hb* is the needle height before compression and *Ha* is the needle height after compression.

##### Loss of Mass While Drying

Microneedles were weighed before and after casting. Following the drying process, the mass loss percentage of each DMN was determined using the equations listed in Ramadon et al. [45], namely:

(4) $$\mathrm{Loss}\;\mathrm{of}\;\mathrm{mass}\;(\%)=\frac{Mw-Ma}{Mw}\;\times \;100\%\;$$

where *Mw* is the mass of the mixture poured into the mold and *Ma* is the dry mass of the dissolving microneedle.

##### Insertion Ability Study

The insertion ability study was carried out with reference to Larrañeta et al. [43] using eight layers of Parafilm^®^ M as an artificial skin model. The DMN prepared was applied manually into eight layers of Parafilm^®^ M using the applicator and pressing with the thumb for 30 s. After insertion, each layer of Parafilm^®^ M was visualized under a digital microscope, and then the number of holes formed by DMN in each layer was counted and recorded [45].

(5) $$\mathrm{Hole}\;\mathrm{formed}\;(\%)=\frac{many\;holes\;formed}{total\;of\;all\;needles}\;\times \;100\%$$

##### In-Skin Dissolution Study

An in-skin dissolution study was carried out using applied rat skin with DMN. The skin used in this study was the skin of Sprague Dawley female rats weighing 200–250 kg. The rat was sacrificed by administering an excess dose of ether, and their breath was cut off by pressing on the neck. Next, the rat’s fur was shaved on the abdominal part using a hair clipper. The skin of the rat was then incised and degreased in the subcutaneous area carefully. The incised skin was stored for 30 min in PBS at pH 7.4 and then stored at 4 °C. The method of sacrificing rats is carried out in accordance with applicable ethics guidance, and for this study, a certificate of ethical approval was obtained from the Ethics Committee of the Faculty of Medicine, University of Indonesia, with letter number KET-487/UN2.F1/ETIK/PPM.00.02/2022. The prepared skin was used within 24 h [50]. Rat skin samples were stored in closed Petri dishes at −20 °C until used. Prior to use, the skin samples were shaved and conditioned in a phosphate-buffered solution (PBS) (pH 7.4) for 15 min before use. DMN was applied to the surface of the rat skin using an applicator with manual pressure for 30 s. A 5.0 g metal cylinder was placed on the top of the microneedle array to prevent microneedle movement. At the predetermined time points, the microneedle array was pulled out from the skin, and the changes were seen using a digital microscope [45].

##### Determination of Ketoprofen Content in Dissolving Microneedles

The determination of ketoprofen content was carried out on the gel solution before the casting process, dissolving the microneedle array after drying on day 0 and dissolving the microneedle array after drying on day 30. Samples were placed in an airtight container and stored at room temperature. Each sample was dissolved in 1 mL of a mixture of acetonitrile and phosphate buffer (pH 3.5) (50:50) in a glass bottle at 37 °C and then vortexed. After dissolving, the sample was then filtered using a 0.22 m membrane filter and diluted for analysis using an HPLC instrument, as described in Section 3.2.9 [22,45].

#### 3.2.8. Differential Scanning Calorimetry (DSC) Analysis of DMN

Differential scanning calorimetry of the selected DMN formulas was carried out using the same method as mentioned in Section Differential Scanning Calorimetry (DSC) Analysis of Co-grinded Ketoprofen. As for the selected NS formulation (PVA 2%), before the DSC analysis, the solution was placed into a temperature-controlled freezer at −80 °C for 3 h prior to lyophilization [48]. Lyophilization was carried out using a freeze drier (VirtisTM Advantage XL-70, SP Scientific, Warminster, PA, USA).

#### 3.2.9. In Vitro Permeation Study

An in vitro permeation test was carried out using Franz diffusion cells. Rat skin was obtained using the same method as described in Section In Skin Dissolution Study. The skin was placed onto the donor compartment with the stratum corneum facing upwards. DMN was inserted into the center of the rat skin for 30 s using an applicator, and a metal weight (5 g) was placed on top of it. The temperature was maintained at 37 °C ± 1 °C, and the receptor compartment was rotated at a speed of 600 rpm. Samples (200 μL) were taken at predetermined time intervals, namely 0,25, 0,5, 0,75, 1, 2, 3, 4, 6, 8, 10, 12, and 24 h, then immediately replaced with fresh PBS pH 7.4. All samples were analyzed using the HPLC method, as described in Section 3.2.9 [22,38].

## 4. Conclusions

This paper, for the first time, successfully developed DMN formulations containing nanosuspensions and Co-grinded ketoprofen. Based on the results of the study, it can be concluded that the solubility properties of ketoprofen can be improved by formulating this drug into such systems. Moreover, this research also provided the proof of concept of using a combination of DMN with nanosuspension and Co-grinded systems for delivering ketoprofen transdermally, particularly as a promising strategy to obtain a sustained-release system. In the future, an in vivo permeation study of DMN containing ketoprofen combined with either nanosuspension or Co-grinded technology using an animal model should also be performed to confirm the ability of these systems to enhance the bioavailability of ketoprofen delivered via the transdermal route.

## Figures and Tables

**Table 1:** Coefficient of determination (R^2^) of the calibration curves, limit of detection (LOD), and limit of quantification (LOQ) of the HPLC method for ketoprofen.

Range (µg/mL)	Slope	Intercept	R^2^	LOD (µg/mL)	LOQ (µg/mL)
5–30	1.7569	−0.0257	0.9995	0.7056	2.1382

**Table 2:** Particle Size Distribution and Zeta Potential of Nanosuspension Formulations (n = 3, mean ± SD).

Formulations	PVA (%)	Dv-90 (nm)	PDI	Zeta Potential (mV)
NS-1	0.5	578.67 ± 30.82	0.467 ± 0.07	−19.17 ± 3.70
NS-2	1	256.33 ± 44.07	0.471 ± 0.11	−17.63 ± 1.90
NS-3	2	124.3 ± 26.15	0.265 ± 0.02	−15.7 ± 3.72

PVA: polyvinyl alcohol; PDI: polydispersity index.

**Table 3:** Formulations of Co-grinded Ketoprofen.

Formulation	Composition
CG-1	K:P = 3:1
CG-2	K:P = 5:1
CG-3	K:P = 10:1
CG-4	K:A = 1:1
CG-5	K:A = 1:2
CG-6	K:A = 2:1

K: Ketoprofen; P: polyvinyl pyrrolidone; A: polyvinyl alcohol.

**Table 4:** Observation Results of Dissolving Microneedle Loaded with Ketoprofen Nanosuspension Through a Microscope (scale bar: 500 μm).

Formulations	Composition	Morphology	
F1-MN NS	PVA 10%	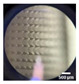	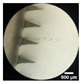
F2-MN NS	PVP 30%	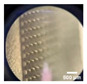	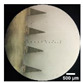
F3-MN NS	PVA 5% PVP 5%	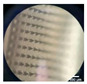	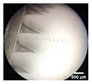
F4-MN NS	PVA 5% PVP 10%	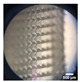	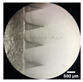
F5-MN NS	PVA 5% PVP 15%	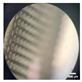	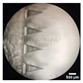

PVA: polyvinyl alcohol; PVP: polyvinyl pyrrolidone.

**Table 5:** Observation Results of Dissolving Microneedle Loaded with Co-grinded Ketoprofen Through a Microscope (scale bar: 500 μm). (PVA: polyvinyl alcohol; PVP: polyvinyl pyrrolidone; Keto: Ketoprofen).

Formulations	Composition	Morphology	
F1-MN CG	Co-grinded Keto 25% PVA 10%	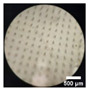	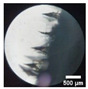
F2-MN CG	Co-grinded Keto 25% PVA 30%	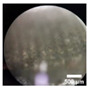	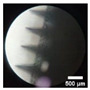
F3-MN CG	Co-grinded Keto 25% PVA 2.5% PVP 5%	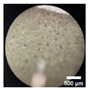	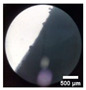
F4-MN CG	Co-grinded Keto 25% PVA 2.5% PVP 10%	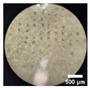	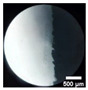
F5-MN CG	Co-grinded Keto 25% PVA 2.5% PVP 15%	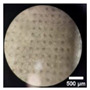	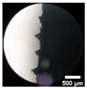
F6-MN CG	Co-grinded Keto 25% PVA 5% PVP 5%	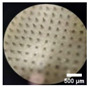	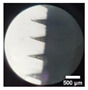
F7-MN CG	Co-grinded Keto 25% PVA 5% PVP 10%	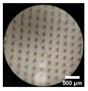	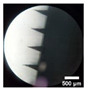
F8-MN CG	Co-grinded Keto 25% PVA 5% PVP 15%	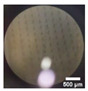	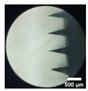
F9-MN CG	Co-grinded Keto 25% PVA 7.5% PVP 5%	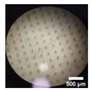	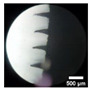
F10-MN CG	Co-grinded Keto 25% PVA 7.5% PVP 10%	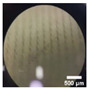	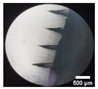
F11-MN CG	Co-grinded Keto 25% PVA 7.5% PVP 15%	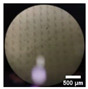	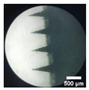

**Table 6:** Height Reduction in DMN Containing Nanosuspension and Co-grinded Ketoprofen (n = 3, mean ± SD).

Formulations	Polymer	Height Reduction (%) ± SD
F1-MN NS	PVA 10%	13.33 ± 1.655
F2-MN NS	PVA 30%	18.989 ± 2.935
F3-MN NS	PVA 5% PVP 5%	14.57 ± 0.631
F4-MN NS	PVA 5% PVP 10%	12.06 ± 0.481
F5-MN NS	PVA 5% PVP 15%	4.239 ± 0.452
F6-MN CG	PVA 5% PVP 5%	6.77 ± 2.79
F7-MN CG	PVA 5% PVP 10%	2.38 ± 1.52
F8-MN CG	PVA 5% PVP 15%	0.58 ± 0.21
F9-MN CG	PVA 7.5% PVP 5%	20.89 ± 1.17
F10-MN CG	PVA 7.5% PVP 10%	6.20 ± 0.45
F11-MN CG	PVA 7.5% PVP 15%	1.26 ± 0.56

PVA: polyvinyl alcohol; PVP: polyvinyl pyrrolidone.

**Table 7:** Loss of Mass of DMN Containing Nanosuspension and Co-grinded Ketoprofen (n = 3, mean ± SD).

Formulations	Polymer	Loss of Mass (%) ± SD
F1-MN NS	PVA 10%	88.10 ± 0.063
F3-MN NS	PVA 5% PVP 5%	92.16 ± 2.937
F4-MN NS	PVA 5% PVP 10%	79.56 ± 0.410
F5-MN NS	PVA 5% PVP 15%	79.24 ± 0.392
F6-MN CG	PVA 5% PVP 5%	71.26 ± 0.084
F7-MN CG	PVA 5% PVP 10%	67.50 ± 0.58
F8-MN CG	PVA 5% PVP 15%	61.79 ± 1.051
F9-MN CG	PVA 7.5% PVP 5%	65.66 ± 1.17
F10-MN CG	PVA 7.5% PVP 10%	59.50 ± 0.365
F11-MN CG	PVA 7.5% PVP 15%	54.53 ± 1.144

PVA: polyvinyl alcohol; PVP: polyvinyl pyrrolidone.

## Data Availability

Not applicable.

## References

[1] Kumar V., Abbas A., Aster J. (2017). Robbins and Cotran Pathologic Basis of Disease.

[2] Sasono B., Amanda N.A., Dewi D.N.S.S. (2020). Faktor Dominan pada Penderita Osteoarthritis di RSUD dr. Mohamad Soewandhie, Surabaya, Indonesia. J. Med. Udayana..

[3] Kemenkes R.I. (2018). Hasil Riset Kesehatan Dasar Tahun 2018. Kementrian Kesehat RI.

[4] Sweetman S.C. (2009). Martindale The Complete Drug Reference 36th Edition. J. Chem. Inf. Model..

[5] Vučen S.R., Vuleta G., Crean A.M., Moore A.C., Ignjatovic N., Uskokovic D. (2013). Improved percutaneous delivery of ketoprofen using combined application of nanocarriers and silicon microneedles. J. Pharm. Pharmacol..

[6] Vonkeman H.E., van de Laar M.A.F.J. (2010). Nonsteroidal Anti-Inflammatory Drugs: Adverse Effects and Their Prevention. Semin. Arthritis Rheum..

[7] Ramadon D., McCrudden M.T.C., Courtenay A.J., Donnelly R.F. (2021). Enhancement strategies for transdermal drug delivery systems: Current trends and applications. Drug Deliv. Transl. Res..

[8] Manasco J.L., Tang C., Burns N.A., Saquing C.D., Khan S.A. (2014). Rapidly dissolving poly(vinyl alcohol)/cyclodextrin electrospun nanofibrous membranes. RSC Adv..

[9] Fukushima K., Ise A., Morita H., Hasegawa R., Ito Y., Sugioka N., Takada K. (2010). Two-Layered Dissolving Microneedles for Percutaneous Delivery of Peptide/Protein Drugs in Rats. Pharm. Res..

[10] Dragicevic N., Maibach H.I. (2016). Percutaneous Penetration Enhancers Chemical Methods in Penetration Enhancement: Nanocarriers.

[11] Mahato R. (2017). Microneedles in Drug Delivery. Emerging Nanotechnologies for Diagnostics, Drug Delivery and Medical Devices.

[12] Larrañeta E., Lutton R.E.M., Woolfson A.D., Donnelly R.F. (2016). Microneedle arrays as transdermal and intradermal drug delivery systems: Materials science, manufacture and commercial development. Mater. Sci. Eng. R Rep..

[13] Duarah S., Sharma M., Wen J. (2019). Recent advances in microneedle-based drug delivery: Special emphasis on its use in paediatric population. Eur. J. Pharm. Biopharm..

[14] Lahiji S.F., Dangol M., Jung H. (2015). A patchless dissolving microneedle delivery system enabling rapid and efficient transdermal drug delivery. Sci. Rep..

[15] Dangol M., Yang H., Li C.G., Lahiji S.F., Kim S., Ma Y., Jung H. (2016). Innovative polymeric system (IPS) for solvent-free lipophilic drug transdermal delivery via dissolving microneedles. J. Control. Release.

[16] Roberts M., Mohammed Y., Pastore M., Namjoshi S., Yousef S., Alinaghi A., Haridass I., Abd E., Leite-Silva V., Benson H. (2017). Topical and cutaneous delivery using nanosystems. J. Control. Release.

[17] Hilaliyati N., Ben E.S., Zaini E. (2017). Enhanced Dissolution Rate of Ketoprofen by Co-grinding Technique with Hydroxypropyl Methylcellulose E6 polymer. J. Sains Farm Klin..

[18] Garg A., Singh S., Rao V.U., Bindu K., Balasubramaniam J. (2009). Solid State Interaction of Raloxifene HCl with Different Hydrophilic Carriers During Co-grinding and its Effect on Dissolution Rate. Drug Dev. Ind. Pharm..

[19] Hassan A., Shantier S.W.G.-K. (2019). Development and Validation of UV-Visible Spectrophotometric Method for Estimation of Ketoprofen in Capsule and Tablet Dosage. Indo. Am. J. Pharm. Res..

[20] ICH International Council for Harmonisation of Technical Requirements for Pharmaceuticals for Human Use (ICH), Validation of Analytical Procedures: Text and Methodology Q2 (R2). https://www.ema.europa.eu/en/documents/scientific-guideline/ich-guideline-q2r2-validation-analytical-procedures-step-2b_en.pdf.

[21] Ferraz-Carvalho R., Mendonça E.A.M., Silva J.P.A., Cavalcanti I.M.F., Galdino S.L., Pitta I.R., Lima M.D.C.A., Santos-Magalhães N.S., Lira-Nogueira M.C.B. (2015). Validation of a UV-spectrophotometric analytical method for determination of LPSF/AC04 from inclusion complex and liposomes. Braz. J. Pharm. Sci..

[22] Chourasia M.K., Kang L., Chan S.Y. (2011). Nanosized ethosomes bearing ketoprofen for improved transdermal delivery. Results Pharma Sci..

[23] European Medicine Agency (2011). Guideline On Bioanalytical Method Validation. https://www.ema.europa.eu/en/documents/scientific-guideline/guideline-bioanalytical-method-validation_en.pdf.

[24] FDA (2016). Analytical Method Validation. New Drug Development: Regulatory Paradigms for Clinical Pharmacology and Biopharmaceutics.

[25] Vora L., Vavia P.R., Larrañeta E., Bell S.E., Donnelly R.F. (2018). Novel nanosuspension-based dissolving microneedle arrays for transdermal delivery of a hydrophobic drug. J. Interdiscip. Nanomed..

[26] Chonkar A.D., Rao J.V., Managuli R.S., Mutalik S., Dengale S., Jain P., Udupa N. (2016). Development of fast dissolving oral films containing lercanidipine HCl nanoparticles in semicrystalline polymeric matrix for enhanced dissolution and ex vivo permeation. Eur. J. Pharm. Biopharm..

[27] Bartos C., Jójárt-Laczkovich O., Katona G., Budai-Szűcs M., Ambrus R., Bocsik A., Gróf I., Deli M.A., Szabó-Révész P. (2018). Optimization of a combined wet milling process in order to produce poly(vinyl alcohol) stabilized nanosuspension. Drug Des. Dev. Ther..

[28] Wada K. (2006). FTIR Talk Letter. Shimadzu. https://www.shimadzu.eu/ftir-talk-letter-archive.

[29] Wicaksono Y., Setyawan D., Siswandono S. (2018). Multicomponent Crystallization of for Improving the Solubility. Chem. J. Mold..

[30] Chan S.-Y., Chung Y.-Y., Cheah X.-Z., Tan E.Y.-L., Quah J. (2015). The characterization and dissolution performances of spray dried solid dispersion of ketoprofen in hydrophilic carriers. Asian J. Pharm. Sci..

[31] Halim A., Hamdeni S., Zaini E. (2013). Peningkatan Laju Disolusi Trimetoprim dengan Teknik Co-Grinding Menggunakan Polimer Polivinilpirolidon K-30 (Enhanced Dissolution Rate of Trimethoprim by Co-grinding Technique with Polyvinylpyrrolidone K-30 Polymer). J. Ilmu. Kefarmasian Indones..

[32] Thomas D., Zhuravlev E., Wurm A., Schick C., Cebe P. (2018). Fundamental thermal properties of polyvinyl alcohol by fast scanning calorimetry. Polymer.

[33] Chauhan A. (2014). Powder XRD Technique and its Applications in Science and Technology. J. Anal. Bioanal. Tech..

[34] Browne E., Worku Z.A., Healy A.M. (2020). Physicochemical Properties of Poly-vinyl Polymers and Their Influence on Ketoprofen Amorphous Solid Dispersion Performance: A Polymer Selection Case Study. Pharmaceutics.

[35] Ardiansyah S., Nasrul E., Rivai H., Ben E.S., Zaini E. (2015). Physicochemical Physicochemical characterization of amorphous solid dispersion of ketoprofen–polyvinylpyrrolidone K-30. Int. J. Pharm. Pharm. Sci..

[36] Nada A., Bandarkar F., Al-basarah Y. (2017). Formulation of Ibuprofen Nanoparticles and Nanosuspensions with Enhanced Dissolution Rate using Ultra-Homogenization Technique. Asian J. Pharm..

[37] Liu T., Wang B., Dong W., Gong J. (2013). Solution-Mediated Phase Transformation of a Hydrate to its Anhydrous Form of Donepezil Hydrochloride. Chem. Eng. Technol..

[38] Permana A.D., Tekko I.A., McCrudden M.T.C., Anjani Q.K., Ramadon D., McCarthy H.O., Donnelly R.F. (2019). Solid lipid nanoparticle-based dissolving microneedles: A promising intradermal lymph targeting drug delivery system with potential for enhanced treatment of lymphatic filariasis. J. Control. Release.

[39] Hutton A.R.J., Quinn H.L., McCague P.J., Jarrahian C., Rein-Weston A., Coffey P.S., Gerth-Guyette E., Zehrung D., Larrañeta E., Donnelly R.F. (2018). Transdermal delivery of vitamin K using dissolving microneedles for the prevention of vitamin K deficiency bleeding. Int. J. Pharm..

[40] Teodorescu M., Bercea M. (2015). Poly(vinylpyrrolidone)—A Versatile Polymer for Biomedical and Beyond Medical Applications. Polym. Plast. Technol. Eng..

[41] Wang Q.L., Ren J.W., Chen B.Z., Jin X., Zhang C.Y., Guo X.D. (2018). Effect of humidity on mechanical properties of dissolving microneedles for transdermal drug delivery. J. Ind. Eng. Chem..

[42] Choudhary S., Sengwa R. (2018). ZnO nanoparticles dispersed PVA–PVP blend matrix based high performance flexible nanodielectrics for multifunctional microelectronic devices. Curr. Appl. Phys..

[43] Larrañeta E., Moore J., Vicente-Pérez E.M., González-Vázquez P., Lutton R., Woolfson A.D., Donnelly R.F. (2014). A proposed model membrane and test method for microneedle insertion studies. Int. J. Pharm..

[44] Putri H.E., Utami R.N., Wahyudin E., Oktaviani W.W., Mudjahid M., Permana A.D. (2021). Dissolving Microneedle Formulation of Ceftriaxone: Effect of Polymer Concentrations on Characterisation and Ex Vivo Permeation Study. J. Pharm. Innov..

[45] Ramadon D., Permana A.D., Courtenay A.J., McCrudden M.T.C., Tekko I.A., McAlister E., Anjani Q.K., Utomo E., McCarthy H.O., Donnelly R.F. (2020). Development, Evaluation, and Pharmacokinetic Assessment of Polymeric Microarray Patches for Transdermal Delivery of Vancomycin Hydrochloride. Mol. Pharm..

[46] González-Vázquez P., Larrañeta E., McCrudden M.T.C., Jarrahian C., Rein-Weston A., Quintanar-Solares M., Zehrung D., McCarthy H., Courtenay A.J., Donnelly R.F. (2017). Transdermal delivery of gentamicin using dissolving microneedle arrays for potential treatment of neonatal sepsis. J. Control. Release.

[47] Nornoo A.O., Wulz J., Yoon H., Nan Y., Lese M. (2014). Impact of the chemical and physical stability of ketoprofen compounded in various pharmaceutical bases on its topical and transdermal delivery. Pharm. Dev. Technol..

[48] Anjani Q.K., Sabri A.H., Castellanos N.M., Utomo E., Martinez A.C., Robles D.J., Wardoyo L.A.H., Donnelly R.F. (2022). Soluplus-based dissolving microarray patches loaded with colchicine: Towards a minimally invasive treatment and management of gout. Biomater. Sci..

[49] Zsikó S., Csányi E., Kovács A., Budai-Szűcs M., Gácsi A., Berkó S. (2019). Methods to Evaluate Skin Penetration In Vitro. Sci. Pharm..

[50] Ramadon D., Anwar E., Harahap Y. (2017). In vitro Penetration and Bioavailability of Novel Transdermal Quercetin-loaded Ethosomal Gel. Indian J. Pharm. Sci..

[51] McGrath M.G., Vucen S., Vrdoljak A., Kelly A., O’Mahony C., Crean A.M., Moore A. (2014). Production of dissolvable microneedles using an atomised spray process: Effect of microneedle composition on skin penetration. Eur. J. Pharm. Biopharm..

[52] Garland M.J., Migalska K., Tuan-Mahmood T.-M., Singh T.R.R., Majithija R., Caffarel-Salvador E., McCrudden C.M., McCarthy H.O., Woolfson A.D., Donnelly R.F. (2012). Influence of skin model on in vitro performance of drug-loaded soluble microneedle arrays. Int. J. Pharm..

[53] Kaleemullah M., Jiyauddin K., Thiban E., Rasha S., Al-Dhalli S., Budiasih S., Gamal O., Fadli A., Eddy Y. (2016). Development and evaluation of Ketoprofen sustained release matrix tablet using Hibiscus rosa-sinensis leaves mucilage. Saudi Pharm. J..

[54] He M., Yang G., Zhang S., Zhao X., Gao Y. (2018). Dissolving Microneedles Loaded with Etonogestrel Microcrystal Particles for Intradermal Sustained Delivery. J. Pharm. Sci..

[55] Du G., Sun X. (2020). Current Advances in Sustained Release Microneedles. Pharm. Front..

[56] Tekko I., Permana A.D., Vora L., Hatahet T., McCarthy H., Donnelly R.F. (2020). Localised and sustained intradermal delivery of methotrexate using nanocrystal-loaded microneedle arrays: Potential for enhanced treatment of psoriasis. Eur. J. Pharm. Sci..

[57] Kim B., Cho H.-E., Moon S.H., Ahn H.-J., Bae S., Cho H.-D., An S. (2020). Transdermal delivery systems in cosmetics. Biomed. Dermatol..

[58] Ameri M., Lewis H., Lehman P. (2018). Effect of Skin Model on In Vitro Performance of an Adhesive Dermally Applied Microarray Coated with Zolmitriptan. J. Pharm..

[59] Iskandarsyah I., Mutakim A. (2010). Preparasi dan Karakterisasi Nanosuspensi dengan Polivinilpirolidon (PVP) Sebagai Pembawa Nanopartikel Senyawa Asam Mefenamat. Pharm. Sci. Res..

[60] Zafar F., Shoaib M.H., Naz A., Yousuf R.I., Ali H. (2013). Determination of Ketoprofen in Human Plasma by RP-HPLC. Am. J. Anal. Chem..

[61] Guo B., Liu H., Li Y., Zhao J., Yang D., Wang X., Zhang T. (2014). Application of phospholipid complex technique to improve the dissolution and pharmacokinetic of probucol by solvent-evaporation and co-grinding methods. Int. J. Pharm..

[62] Watson D. (2020). Pharmaceutical Analysis: A Textbook for Pharmacy Students and Pharmaceutical Chemists.

[63] Yadav P.S., Kumar V., Singh U.P., Bhat H.R., Mazumder B. (2011). Physicochemical characterization and in vitro dissolution studies of solid dispersions of ketoprofen with PVP K30 and d-mannitol. Saudi Pharm. J..

[64] Rosaini H., Sari Y.E.N., Makmur I., Halim A., Sidoretno W.M. (2020). Karakterisasi Sifat Fisikokimia Sistem Dispersi Padat Nimodipin Dengan Poloxamer 188 Menggunakan Metode Penggilingan Bersama. JOPS J. Pharm. Sci..

[65] Khaleel N.Y., Abdulrasool A.A., Ghareeb M.M., Hussain S.A. (2011). Solubility and dissolution improvement of ketoprofen by solid dispersion in polymer and surfactant using solvent evaporation method. Int. J. Pharm. Pharm. Sci..

[66] Ramadon D., Sutrisna L.F.P., Harahap Y., Putri K.S.S., Ulayya F., Hartrianti P., Anjani Q.K., Donnelly R.F. (2023). Enhancing Intradermal Delivery of Lidocaine by Dissolving Microneedles: Comparison between Hyaluronic Acid and Poly(Vinyl Pyrrolidone) Backbone Polymers. Pharmaceutics.

[67] Abdelghany S., Tekko I.A., Vora L., Larrañeta E., Permana A.D., Donnelly R.F. (2019). Nanosuspension-Based Dissolving Microneedle Arrays for Intradermal Delivery of Curcumin. Pharmaceutics.

